# Bioconversion of selenite to selenium nanoparticles by *Paenibacillus polymyxa* P10: metabolic basis and potential for wastewater treatment

**DOI:** 10.1128/aem.00704-26

**Published:** 2026-07-17

**Authors:** Jiaxing Zhou, Changpeng Sang, Zhunan Liu, Weiming Li, Yanchen Sun, Jiaxi Zhang, Shaofeng Wang, Xiangfeng Zeng

**Affiliations:** 1 Key Laboratory of Industrial Ecology and Environmental Engineering (Ministry of Education), School of Environmental Science and Technology, Dalian University of Technology, Dalian, China; 2 Institute of Applied Ecology, Chinese Academy of Sciences 74763, Shenyang, Liaoning, China; 3 Liaoning Geology and Mineral Resources Institute Co., Ltd., Shenyang, China; 4 Department of Marine Chemistry and Geochemistry, Woods Hole Oceanographic Institution 10627, Woods Hole, Massachusetts, USA; University of Minnesota Twin Cities, St. Paul, Minnesota, USA

**Keywords:** selenium reduction, resource recovery, *Paenibacillus polymyxa *P10, high-concentration tolerance, nitrite reductase

## Abstract

Sustainable bioprocesses for selenite [Se(IV)] remediation and selenium (Se) recovery are constrained by the limited tolerance of most reported microorganisms for elevated Se(IV) concentrations. Here, we identified *Paenibacillus polymyxa* P10, isolated from estuarine sediment, as a highly tolerant and efficient Se(IV)-reducing bacterium. Strain P10 completely reduced 5 mM Se(IV) to Se nanoparticles (SeNPs) within 48 h and retained measurable Se(IV)-reducing activity up to 1,000 mM Se(IV). It also maintained activity over a wide range of pH (5–10), temperature (20–45°C), and salinity (1%–3.5%) conditions. In addition, P10 maintained high Se(IV) reduction performance over five repeated-batch cycles. Transcriptomic and enzymatic analyses showed that Se(IV) exposure triggered coordinated metabolic responses, including carbohydrate metabolism, oxidative phosphorylation, and nitrogen metabolism, which together supported energy generation and reductive transformation. Nitrite reductase-associated activity was linked to Se(IV) reduction. These findings establish strain P10 as a robust biocatalyst for Se(IV) detoxification and SeNP recovery, and provide mechanistic insight into microbial selenite reduction relevant to the development of scalable Se-containing wastewater treatment processes.

**IMPORTANCE:**

Selenite [Se(IV)] is among the most problematic selenium (Se) species in wastewater due to its high toxicity and mobility. Although microbial Se(IV) reduction is an attractive strategy for Se detoxification and recovery, its practical use is limited by the poor tolerance and weak environmental robustness of most known microorganisms. This study identifies a highly tolerant Se(IV)-reducing bacterium with broad adaptability to pH, temperature, and salinity stress, extending the known ecological and functional potential of microbial Se reducers. These findings strengthen the basis for developing more reliable bioprocesses for Se-contaminated wastewater treatment and Se resource recovery.

## INTRODUCTION

Selenium (Se) is an essential trace element for humans, but the margin between requirement and toxicity is narrow (1, 2). Excessive Se exposure can induce oxidative stress and genotoxic effects, making Se contamination a growing environmental concern, particularly in industrial wastewaters (3). Regulatory limits for Se in wastewater and drinking water further highlight the need for efficient treatment technologies. The U.S. Environmental Protection Agency (EPA) mandates a discharge limit of 5 μg Se/L for Se-containing wastewater (4), while several nations have implemented a maximum threshold of 50 μg Se/L for drinking water (5). Among inorganic Se species, selenite [Se(IV)] is of particular concern because of its high solubility, bioavailability, and toxicity (6, 7). By contrast, reduction of Se(IV) to elemental Se [Se(0)] decreases Se mobility and bioavailability and simultaneously generates a recoverable product with potential applications in electronics, medicine, and other technologies (7, 8).

Se concentrations in industrial wastewaters can far exceed natural background levels, with reported levels ranging from 0.4 to 53 mg/L, and Se(IV) concentrations in acidic mine drainage, hydrometallurgical effluents, and engineered bioreactors commonly reaching 0.1–8 mM (9–11). Such concentrations indicate that practical treatment systems must remain effective under high Se(IV) loading rather than only under dilute laboratory conditions. Although various physicochemical methods have been used for Se(0) synthesis, such as thermal decomposition, coprecipitation, electrochemical, and photochemical approaches (5), these methods often involve high operational costs, harsh reaction conditions, and the potential risk of secondary pollution (10, 12).

Microbial reduction offers an attractive alternative because Se oxyanions can be transformed under relatively mild conditions and immobilized as elemental Se nanoparticles. Previous studies have further emphasized that biological Se treatment is attractive not only for detoxification but also for Se recovery from industrial waste streams (7, 13). However, the performance of reported Se(IV)-reducing bacteria remains highly variable under elevated Se(IV) stress and changing environmental conditions. For example, *Bacillus safensis* JG-B5T exhibited pronounced cell deformation at 2.5 mM Se(IV), *Stenotrophomonas maltophilia* SeITE02 showed reduced cell yield with increasing Se(IV) concentration, and the Se(IV)/Se(VI) reduction efficiency of *Pseudomonas stutzeri* NT-I decreased by 60% when salinity increased from 0.5% to 2% (13–15). Although several highly tolerant strains have been reported, including *Providencia rettgeri* HF16-A and *Proteus mirabilis* QZB-2 (16, 17), previous studies have mainly focused on tolerance thresholds and reduction capacity. Less is known about whether highly tolerant strains can maintain reduction efficiency across broad pH, salinity, and temperature ranges or during repeated operation, all of which are important for engineered wastewater treatment. In addition, the organisms, pathways, and product characteristics involved in Se oxyanion reduction remain insufficiently resolved in complex wastewater systems.

To address these challenges, *Paenibacillus polymyxa* P10 was isolated from estuarine sediment as a Se(IV)-reducing bacterium with the potential to convert high concentrations of Se(IV) to Se(0). This study systematically investigated strain P10 in terms of Se(IV) reduction kinetics, high-concentration Se(IV) response, environmental adaptability, repeated-batch performance, SeNP formation, and potential detoxification mechanisms. Product characterization, transcriptomic analysis, inhibitor experiments, and enzyme activity assays were combined to evaluate whether nitrogen-metabolism-related pathways, including nitrite reductase-associated processes, may contribute to Se(IV) transformation. This study provides a comprehensive evaluation of a highly Se(IV)-converting *Paenibacillus* strain and offers theoretical foundations and technical insights for developing efficient and sustainable bioremediation technologies for Se recovery.

## RESULTS AND DISCUSSION

### Isolation and identification of Se(IV)-reducing strain P10

A Se(IV)-reducing bacterial strain, designated P10, was successfully isolated from estuarine sediments through an enrichment culture with 5 mM Se(IV). Strain P10 is a Gram-positive bacterium that appears as a rodlike microorganism under a microscope (Fig. S1a). The culture of the isolate showed milky-white broth on a normal medium while turning distinctly reddish when Se(IV) was supplemented, indicating the formation of Se(0) (Fig. S1b). The 16S rRNA gene sequence analysis showed that strain P10 shared 99.9% sequence similarity with *P. polymyxa* type strain. Phylogenetic analysis revealed that strain P10 formed a coherent branch with *P. polymyxa* strain M-1 (Fig. S1c). Combined with morphological and physiological characteristics, strain P10 was identified as *P. polymyxa. P. polymyxa* P10 has been deposited in the China General Microbiological Culture Collection Center (CGMCC; No. 30295). *P. polymyxa* is widely recognized for its diverse ecological functions, including the production of antimicrobial compounds and heavy metal biosorption capabilities (18). The identification of strain P10’s remarkable Se(IV)-reducing ability not only expands the known metabolic versatility of *P. polymyxa* but also provides new insights into bacterium-mediated Se cycling in estuarine ecosystems, particularly in environments with elevated Se concentrations.

### Environmental adaptability and Se(IV) reduction under different conditions

The environmental adaptability of strain P10 was first evaluated in the absence of Se(IV). As shown in Fig. S2a, P10 maintained active growth across a wide pH range (5–10), with optimal growth at pH 7–8. Considering its environmental relevance, pH 7 was selected for subsequent experiments. Temperature profiling showed that P10 grew between 20°C and 45°C, with the highest growth rate at 37°C (Fig. S2b). The strain also tolerated NaCl concentrations of 1–3.5%, while growth was inhibited at 5% salinity (Fig. S2c). These results indicate that strain P10 has broad physiological adaptability, which may be related to extracellular polysaccharides of *P. polymyxa* that contribute to biofilm formation and stress tolerance (18).

**Fig 1:**
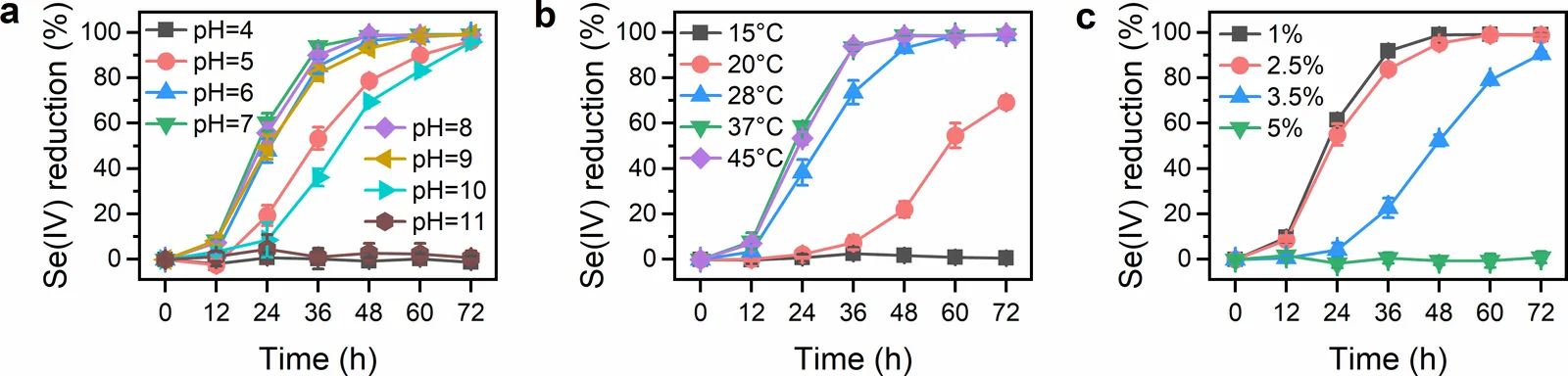
Se(IV) reduction by *P. polymyxa* P10 in modified LB medium under varying conditions: (**a**) different pH levels at 37°C; (**b**) different temperatures at pH 7; and (**c**) different salinities at pH 7 and 37°C. Data are presented as the mean ± SD (*n* = 3).

To further verify whether this adaptability supports Se(IV) bioremediation, Se(IV) reduction was examined under different environmental conditions in the presence of 1 mM Se(IV). Strain P10 retained Se(IV)-reducing activity over a broad pH range, with rapid reduction at pH 7–9 and delayed but substantial reduction at pH 5, 6, and 10 (fig. 1a). Little Se(IV) reduction occurred at pH 4 or 11, indicating that strongly acidic or alkaline conditions suppressed Se(IV) transformation. Compared with many reported Se(IV)-reducing bacteria that are mainly characterized near-neutral pH (6, 13, 16), P10 showed relatively broad pH robustness. Temperature experiments showed that efficient Se(IV) reduction occurred at 28–45°C, particularly at 37°C and 45°C, whereas reduction was delayed at 20°C and negligible at 15°C (Fig. 1b). Under different salinity conditions, P10 showed nearly complete Se(IV) reduction at 1% and 2.5% NaCl and retained substantial reduction at 3.5% NaCl, while 5% NaCl almost completely inhibited reduction (Fig. 1c). This performance contrasts with *P. stutzeri* NT-I, whose Se(IV)/Se(VI) reduction efficiency decreased markedly as salinity increased from 0.5% to 2% (15). Overall, these results demonstrate that strain P10 not only grows under variable environmental conditions but also maintains Se(IV)-reducing capability across a relatively broad range of pH, temperature, and salinity conditions, supporting its potential application in Se-containing wastewater treatment. Based on these findings, subsequent experiments were conducted under pH 7 and 37°C.

### Se(IV) tolerance and reduction capability of *P. polymyxa* P10

**Fig 2:**
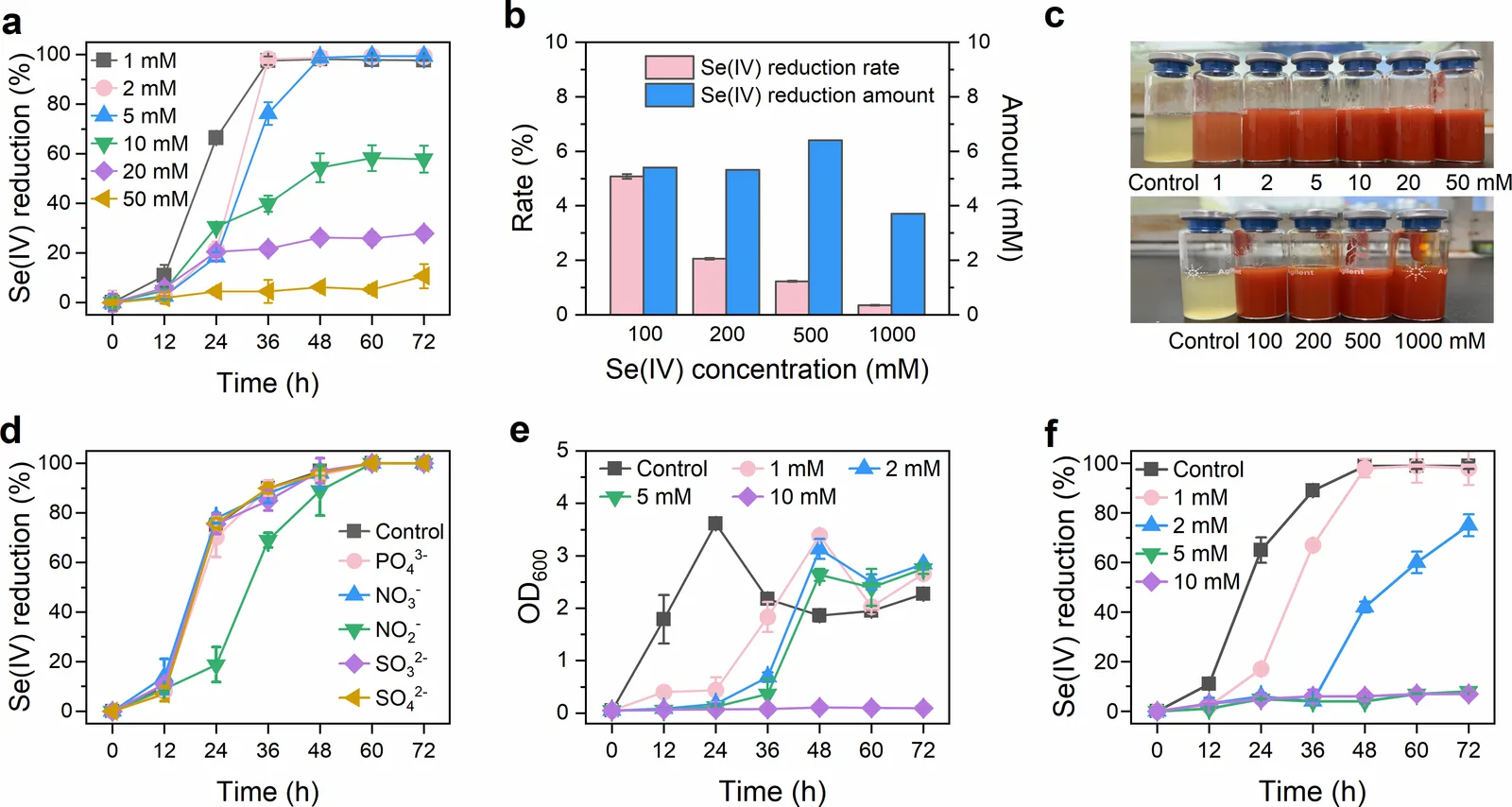
Se(IV) reduction by *P. polymyxa* P10 at pH 7 and 37°C in modified LB medium: (**a**) effects of Se(IV) concentrations from 1 to 50 mM; (**b**) reduction rate and amount at Se(IV) concentrations from 100 to 1,000 mM; (**c**) images of Se(IV) reduction at different concentrations; (**d**) impacts of various anions on 1 mM Se(IV) reduction; (**e**) growth of *P. polymyxa* P10 under different NO_2_^–^ concentrations; and (**f**) Se(IV) reduction of *P. polymyxa* P10 under different NO_2_^–^ concentrations. Data are presented as the mean ± SD (*n* = 3).

*P. polymyxa* P10 was able to grow in medium containing 1–50 mM Se(IV), indicating strong tolerance to elevated Se(IV) concentrations (Fig. 2a). The culture medium began to turn visibly red after 12–24 h of incubation, together with the appearance of a red precipitate, indicating the formation of Se(0) (16). *P. polymyxa* P10 completely reduced 2 mM Se(IV) within 36 h, while 5 mM Se(IV) was completely reduced at 48 h. After 72 h, the reduction rates were 57.9% (10 mM), 27.9% (20 mM), and 10.6% (50 mM), respectively. Here, 1–10 mM Se(IV) was considered relevant to high-Se industrial effluents or concentrated process streams, whereas 20–50 mM Se(IV) was used to evaluate the response of strain P10 under severe Se(IV) stress. The ability of strain P10 to maintain growth and reduction activity under elevated Se(IV) concentrations suggests an efficient detoxification strategy, likely involving the rapid conversion of soluble Se(IV) to insoluble Se(0). The reduced reduction rate at higher Se(IV) levels may be attributed to increased metabolic stress and limited availability of reducing equivalents required for Se(IV) reduction. Therefore, the complete reduction of 2–5 mM Se(IV) and partial reduction of 10–50 mM Se(IV) support the potential of strain P10 for treating high-strength Se-containing wastewater while also demonstrating its resistance to severe Se(IV) stress.

Notably, *P. polymyxa* P10 retained detectable Se(IV)-reducing activity under extremely high initial Se(IV) concentrations, producing a red precipitate even at 100–1,000 mM Se(IV) (Fig. 2b and c). These concentrations are far higher than those in most real wastewater conditions and were therefore used as extreme tolerance-screening conditions rather than engineering-relevant treatment concentrations. Under these conditions, the high-concentration response was evaluated based on visible Se(0) formation and measurable dissolved Se(IV) removal, rather than quantitative growth or viability measurements. Although the reduction efficiency decreased with increasing Se(IV) concentration, the overall reduction pattern remained broadly comparable to that observed at 5–50 mM, with the final Se(IV) concentration decreased by approximately 5 mM. Thus, the 100–1,000 mM tests should be interpreted as evidence of extreme Se(IV) resistance and residual conversion capacity.

This high-concentration response differs from many previously reported bacterial strains, including *Vibrio natriegens* (100 mM) and *Pseudomonas moraviensis* subsp. (120 mM) (19, 20). Although higher tolerance values have recently been reported for *Halomonas* sp. SF2000 (1,200 mM) and *Trichosporon asahii* I30 (2,000 mM), the criteria used to define tolerance were not fully consistent among studies (21, 22). Specifically, the 1,200 mM tolerance of *Halomonas* sp. SF2000 was evaluated from growth on Se(IV)-containing LB agar plates, whereas the 2,000 mM tolerance of *T. asahii* I30 was derived from a 96-well susceptibility assay based mainly on color development, and its growth and reduction kinetics were only further characterized at 0–2 mM Se(IV). Thus, compared with strains for which high Se(IV) resistance was inferred mainly from plate growth, color development, or low-concentration kinetic assays, strain P10 was evaluated based on measurable dissolved Se(IV) removal and visible Se(0) formation across an exceptionally broad range of initial Se(IV) concentrations. This provides a clearer basis for assessing its potential for high-concentration Se(IV) conversion.

The addition of phosphate (PO_4_^3–^), nitrate (NO_3_^–^), sulfite (SO_3_^2–^), and sulfate (SO_4_^2–^) at 1 mM concentration had no significant influence on the Se(IV) reduction by *P. polymyxa* P10 (Fig. 2d). In contrast, nitrite (NO_2_^–^) significantly inhibited Se(IV) reduction, suggesting a potential interaction between nitrite metabolism and Se(IV) reduction pathways. Previous studies have shown that enzymes involved in nitrate or nitrite reduction may also catalyze Se oxyanion reduction (23). Thus, nitrite-related reductases may participate in Se(IV) reduction in strain P10, although further verification is required.

To evaluate the inhibitory effect of nitrite, NO_2_^–^ concentrations ranging from 0 to 10 mM were tested at a fixed Se(IV) concentration of 1 mM. As shown in Fig. 2e and f, at relatively low NO_2_^–^ concentrations, especially 1–2 mM, bacterial growth was only moderately delayed, but Se(IV) reduction was clearly retarded, indicating that NO_2_^–^ may compete with Se(IV) for shared electron donors, electron-transfer components, or reductases involved in oxyanion reduction. At 5 mM NO_2_^–^, bacterial growth was still observable, whereas Se(IV) reduction was almost completely inhibited, further suggesting that the inhibition of Se(IV) reduction cannot be explained solely by reduced biomass accumulation. At 10 mM NO_2_^–^, both bacterial growth and Se(IV) reduction were strongly suppressed, indicating that nitrite toxicity became dominant. Nitrite is known to impair bacterial physiology by affecting aerobic or microaerobic respiration, including heme-copper oxidases and cytochrome c biosynthesis, thereby reducing cellular energy conservation and growth (24). Therefore, the inhibitory effect of NO_2_^–^ on Se(IV) reduction in strain P10 likely resulted from the combined effects of metabolic competition at lower NO_2_^–^ concentrations and physiological toxicity at higher NO_2_^–^ concentrations. In a study on Se(IV) reduction by *Azospirillum brasilense*, Se(IV) reduction was closely correlated with nitrite reduction across the wild-type strain and three derivatives, suggesting that nitrite reductase participates in Se(IV) reduction, although it is unlikely to be the only enzyme involved (25). Our results support a possible association between nitrite reduction pathways and Se(IV) transformation by strain P10, while also indicating that growth inhibition and competition for reductases and electron donors may occur simultaneously.

### Impact of inoculum size on the kinetics of growth and Se(IV) reduction

The effect of inoculum size on the growth of strain P10 and its Se(IV) reduction performance was evaluated using inoculation ratios of 0.5%–5% (vol/vol) (Fig. S3a and b). All treatments showed a short adaptation phase within the first 12 h, followed by rapid growth and accelerated Se(IV) reduction. Increasing the inoculum size clearly enhanced the early-stage reduction kinetics, especially within the first 24–36 h. Inoculum sizes of 2% and 5% achieved faster Se(IV) reduction than the 0.5% and 1% treatments, whereas increasing the inoculum from 2% to 5% provided little additional improvement. After 48 h, nearly complete Se(IV) reduction was observed in all treatments.

These results indicate that inoculum size mainly influences the startup kinetics of Se(IV) reduction rather than the ultimate reduction extent. While low inoculum levels were sufficient for complete Se(IV) removal, moderate inoculum sizes significantly accelerated process initiation. Considering both reduction efficiency and biomass input, a 2% inoculum appears to provide an effective balance for subsequent experiments and potential engineering applications.

### Formation and characterization of SeNPs

**Fig 3:**
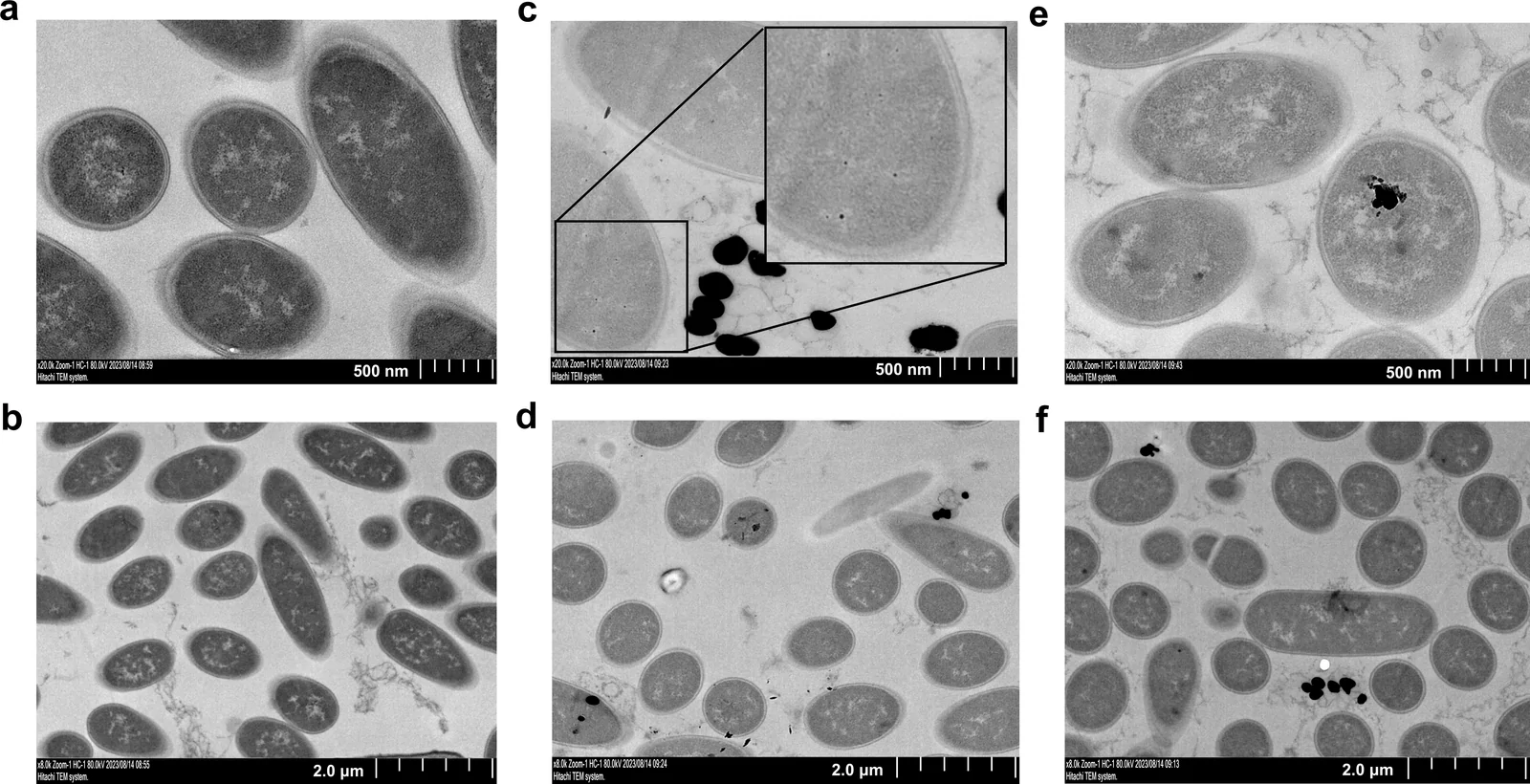
TEM micrographs of *P. polymyxa* P10 and SeNPs: (**a and b**) control after 24 h of incubation; (**c and d**) with 1 mM Se(IV) after 24 h of incubation; and (**e and f**) with 1 mM Se(IV) after 48 h of incubation.

TEM micrographs of *P. polymyxa* P10 cells and reduction products are shown in Fig. 3. No nanoparticles were observed in the control samples (Fig. 3a and b), but nanoparticles can be observed both intracellularly and extracellularly in the Se(IV) treatment. The intracellular nanoparticles had diameters of 10–50 nm at 24 h (Fig. 3c) and 100–150 nm at 48 h (Fig. 3e). This size progression suggests that the Se(IV) reduction by *P. polymyxa* P10 involves a distinct nucleation and growth process. This size progression suggests that Se(IV) reduction by *P. polymyxa* P10 involves a nucleation and growth process. The intracellular occurrence of Se nanoparticles (SeNPs) indicates that at least part of Se(IV) reduction may occur inside the cells. Notably, the diameter of the extracellular nanoparticles is larger than that of the intracellular ones, as shown in Fig. 3c and f.

**Fig 4:**
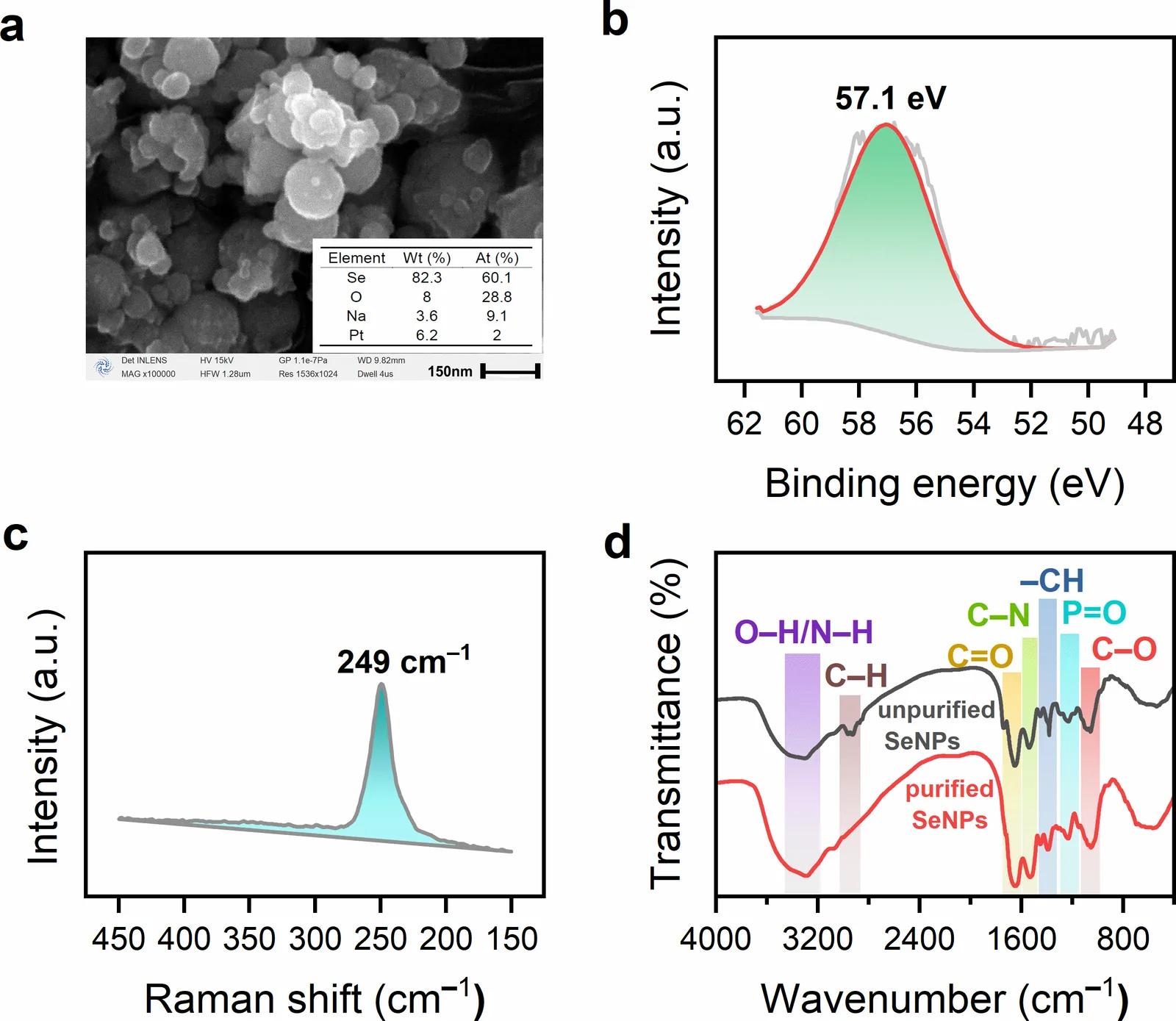
Characterization of SeNPs produced by *P. polymyxa* P10: (**a**) SEM with EDS analysis; (**b**) XPS; (**c**) Raman spectrum; and (**d**) FTIR spectrum.

SEM imaging (Fig. 4a) reveals that the products of Se(IV) reduction by *P. polymyxa* P10 were spherical nanoparticles, with diameters ranging from 50 to 150 nm. EDS analysis showed that the product composition is primarily Se. The size range of SeNPs produced by *P. polymyxa* P10 is similar to that produced by other microorganisms (14, 16). EDS analysis revealed that Se was the dominant element in the microbially synthesized products, with weight and atomic fractions of approximately 82.3% and 60.1%, respectively, while only low levels of impurities such as oxygen and sodium were detected. The red precipitate was analyzed by XPS (Fig. 4b) and confirmed to be SeNPs based on a binding energy position of 57.1 eV (26). The Raman spectrum showed a broad peak at 249 cm^–1^ (Fig. 4c), which directly indicates that the Se-Se vibrations are characteristic of amorphous Se (27, 28).

The FTIR spectrum (Fig. 4d) revealed several characteristic absorption bands. To evaluate whether the purification procedure affected the organic components associated with SeNPs, FTIR spectra of unpurified extracellular SeNPs and purified total SeNPs were compared. The broad band at around 3,300 cm^–1^ was attributed to O–H/N–H stretching vibrations, whereas the band near 1,650 cm^–1^ was assigned to the amide I band, arising mainly from C = O stretching in proteins (16). The peak at 1,530 cm^–1^ was assigned to the amide II band, which was attributed to the stretching vibrations of C–N in proteins (29). The band at 1,400 cm^–1^ was attributed to –CH bending and the existence of C–O or C–H, respectively (16). The band at 1,240 cm^−1^ was assigned to the P = O vibration of phosphodiester located at amide III (29), while the peak centered at 1,046 cm^−1^ may have originated from C–O stretching (30). For the unpurified extracellular SeNPs, an additional absorption band at 2,931 cm**^−^**^1^ was observed, which may correspond to aliphatic C–H stretching vibrations of lipid-like components (30). This band was absent or markedly weakened in the purified SeNPs, suggesting that lipid-like biomolecules were effectively removed during alkaline lysis and n-hexane purification (31). In contrast, the major protein-related bands, particularly the amide I and amide II bands, showed no obvious differences between unpurified extracellular SeNPs and purified SeNPs. This result suggests that proteinaceous components were already associated with extracellular SeNPs during cultivation. Nevertheless, because FTIR analysis cannot fully distinguish tightly bound surface biomolecules from co-precipitated extracellular organic matter, these spectra were interpreted as evidence for biomolecule-related functional groups associated with biogenic SeNPs, rather than as definitive proof of specific surface-bound proteins.

### Global metabolic responses to Se(IV) stress

**Fig 5:**
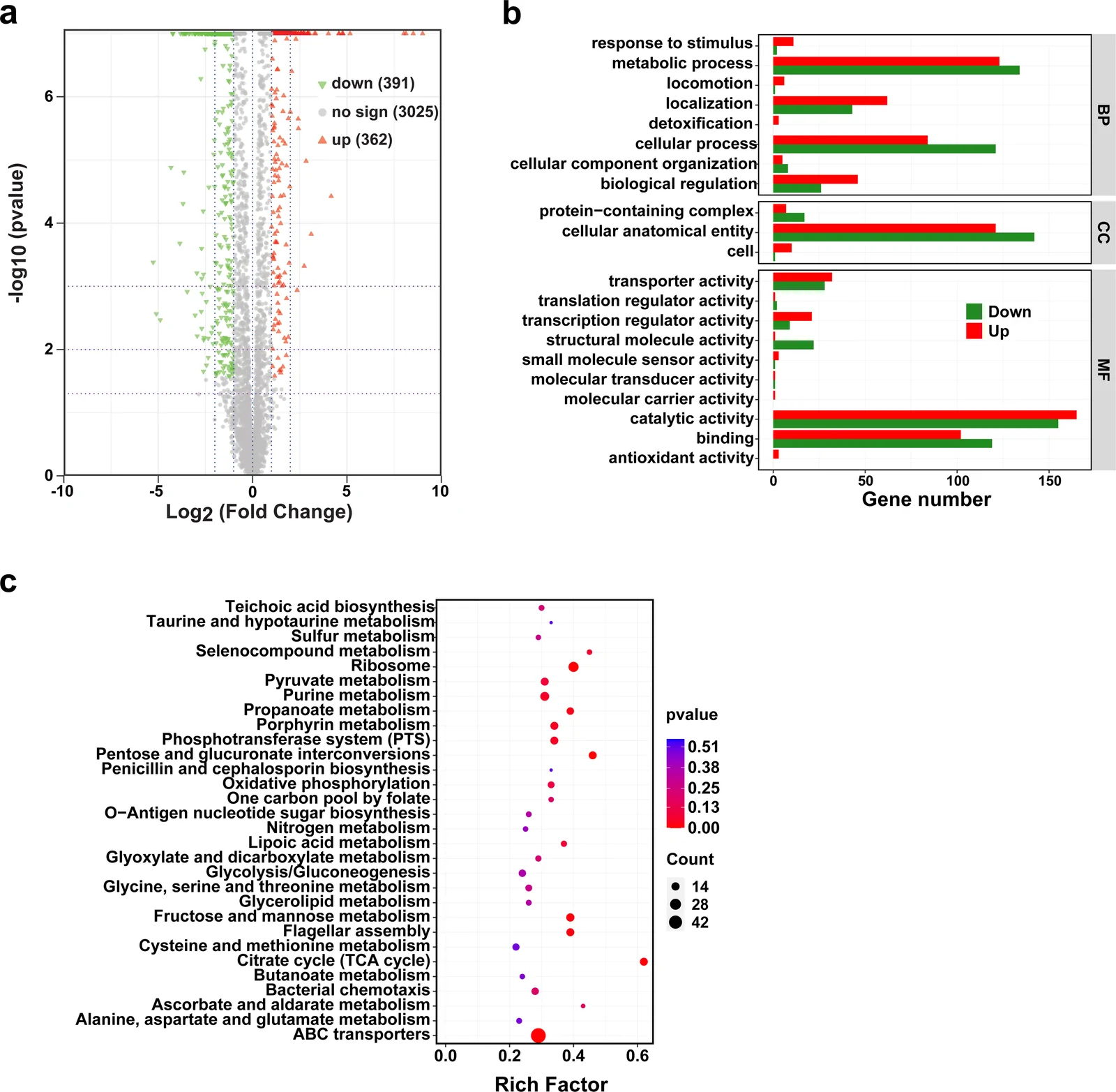
Transcriptome analysis of *P. polymyxa* P10 in response to Se(IV) exposure: (**a**) volcano plot showing up- and downregulated genes; (**b**) GO functional classification of DEGs, including BP (biological process), MF (molecular function), and CC (cellular component); and (**c**) KEGG pathway annotation of the DEGs in the Se(IV) treatment group. RNA-seq was performed with three independent biological replicates per treatment. DEGs were defined as genes with |log_2_FC| ≥ 1 and FDR ≤ 0.05.

Transcriptomic analysis was conducted to investigate the molecular responses of *P. polymyxa* P10 to Se(IV) stress, including tolerance, Se(IV) transformation, and SeNP biosynthesis. Compared to the control, 753 DEGs were identified in the Se(IV) treatment group, with 362 upregulated and 391 downregulated genes (Fig. 5a). GO annotation showed that these DEGs were classified into 21 functional groups, including eight biological processes, three cellular components, and 10 molecular functions (Fig. 5b). The main biological processes involving these DEGs were biological regulation, cellular processes, localization, and metabolic processes. In terms of molecular functions, binding and catalytic activity were the most prevalent terms.

KEGG enrichment (Fig. 5c) indicated that ABC transporters, ribosome, and oxidative phosphorylation were among the pathways most likely relevant to Se(IV) stress response and energy supply (6, 29). The enrichment of ABC transporters may enhance nutrient and ion transport, supporting cellular energy balance under Se(IV) exposure (6). Ribosomal protein enrichment reflects increased protein synthesis, which could contribute to stress adaptation (16). Oxidative phosphorylation likely supplies ATP to maintain cellular metabolism under stress (29).

Other enriched pathways, including carbohydrate metabolism, citrate cycle, porphyrin metabolism, lipoic acid metabolism, and flagellar assembly, may represent general metabolic responses or stress adaptation rather than being directly involved in Se(IV) reduction (32–34). For example, porphyrin metabolism and flagellar assembly have been linked to electron transfer and motility in other Se(IV)-reducing bacteria, suggesting potential but unconfirmed roles in *P. polymyxa* P10 (29, 34). Lipoic acid may help mitigate oxidative stress induced by Se(IV) exposure (34).

Notably, genes associated with selenocompound metabolism, sulfur metabolism, and nitrogen metabolism were enriched, consistent with pathways previously reported to contribute to Se(IV) reduction in *Bacillus subtilis* 168 and *Stenotrophomonas* sp. EGS12 (16, 34, 35). To provide gene-level support for the pathway-level interpretation, representative DEGs from the major Se(IV)-responsive pathways were summarized in Table S1. In nitrogen metabolism, *nirB* encoding nitrite reductase was upregulated (log_2_FC = 1.28, FDR = 1.31 × 10**^−^**^5^), whereas nitrate reductase (*narB*) and a nitrate reductase subunit gene were downregulated, indicating that nitrogen-metabolism-related responses were differentially regulated rather than uniformly activated. In sulfur and selenocompound metabolism, adenylyl-sulfate kinase (*cysC*), cysteine synthase (*cysK*), and thioredoxin reductase (*trxB*) were upregulated, while methionine synthase (*metH*) and 5-methyltetrahydropteroyltriglutamate--homocysteine S-methyltransferase (*metE*) were downregulated, suggesting changes in sulfur assimilation, methionine metabolism, and thioredoxin-dependent redox processes. Several ABC transporter genes, including phosphate ABC transporter permease PstA (*pstA*), phosphate ABC transporter ATP-binding protein PstB (*pstB*), and phosphate ABC transporter permease subunit PstC (*pstC*), were upregulated, whereas oxidative phosphorylation-related genes showed mixed regulation. These gene-level changes support the involvement of transport, redox homeostasis, energy metabolism, and nitrogen/sulfur-related pathways in the Se(IV) stress response of *P. polymyxa* P10.

### Potential role of nitrite reductase in Se(IV) reduction

**Fig 6:**
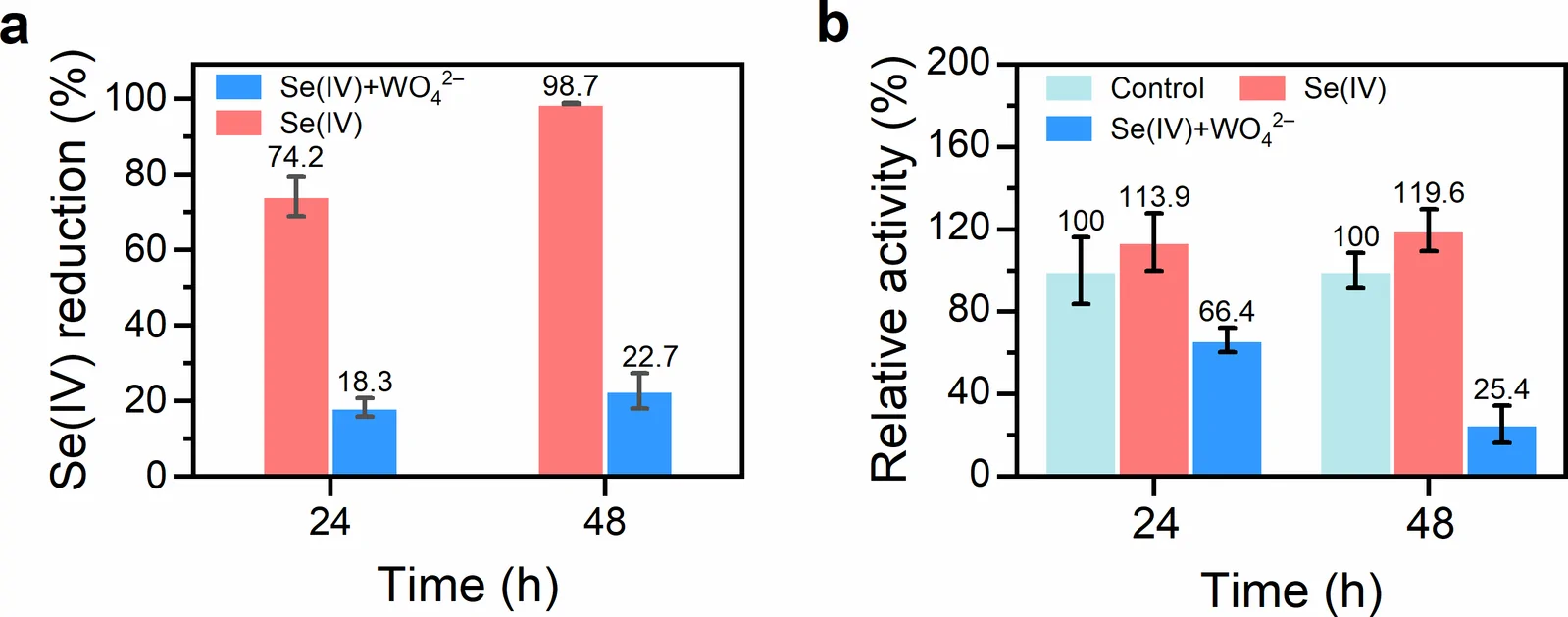
Effects of sodium tungstate on (**a**) Se(IV) reduction and (**b**) nitrite reductase activity of *P. polymyxa* P10 at pH 7 and 37°C in a modified LB medium. (**a**) Se(IV) reduction by *P. polymyxa* P10 under two treatments: without inhibitor and with sodium tungstate. (**b**) Nitrite reductase activity in the two treatment systems, expressed relative to the control (set as 100%). Data are presented as the mean ± SD (*n* = 3).

To evaluate whether nitrite reductase-associated processes contribute to Se(IV) reduction, tungstate inhibition experiments and enzyme activity assays were conducted. Tungstate was used as an inhibitor of molybdenum cofactor-containing enzymes, including nitrate and nitrite reductases. Tungstate treatment strongly inhibited Se(IV) reduction, with the reduction efficiency decreasing from 98.7% in the control to 22.7% at 48 h (Fig. 6a). Although tungstate inhibition alone cannot identify nitrite reductase as the specific Se(IV)-reducing enzyme, it indicates that tungstate-sensitive reductive processes are important for Se(IV) transformation by *P. polymyxa* P10.

Nitrite inhibited Se(IV) reduction, while nitrate had no effect (Fig. 2d), suggesting that nitrite-related reductive metabolism is more closely linked to Se(IV) transformation than nitrate reduction under the tested conditions. The activity of nitrite reductase in *P. polymyxa* P10 was enhanced under Se(IV) stress (Fig. 6b). After 24 and 48 h of the reaction, the nitrite reductase activity was 113.9% and 119.6% of the control treatment, respectively. When tungstate was added, the nitrite reductase activity decreased to 66.4% and 25.4% of the control levels at the corresponding time points. The parallel decline in both nitrite reductase activity and Se(IV) reduction under tungstate treatment suggests that nitrite reductase is likely involved in Se(IV) transformation. These findings align with previous studies on bacterial Se(IV) reduction mechanisms. For instance, nitrite reductase has been identified as the key Se(IV)-reducing enzyme in *Rhizobium sullae* strain HCNT1, where nitrite reductase-deficient mutants completely lost their Se(IV) reduction capability (36).

However, because tungstate can also affect nitrate reductase and other molybdenum-dependent enzymes, the observed inhibition cannot be attributed exclusively to nitrite reductase. The residual 22.7% Se(IV) reduction observed under tungstate inhibition suggests that nitrite reductase-associated processes are important but not exclusive contributors to Se(IV) reduction in strain P10. Other reductases or parallel electron-transfer pathways may also contribute, as reported in bacteria that employ multiple Se(IV) reduction pathways (37).

Overall, our results suggest a possible association between nitrite-reduction-related metabolism and Se(IV) transformation in *P. polymyxa* P10. By linking nitrite inhibition, nitrogen-metabolism-related transcriptional responses, changes in nitrite reductase activity, and tungstate-sensitive reduction phenotypes in a single highly Se(IV)-converting *Paenibacillus* strain, this study provides integrated evidence that nitrite reductase-associated processes may contribute to Se(IV) reduction. However, nitrite reductase should not be regarded as the sole Se(IV)-reducing enzyme, and further biochemical and purified-enzyme analyses are still required to establish its specific catalytic role. These findings provide a basis for future mechanistic studies and for the optimization of enzyme function and metabolic pathways in strain P10.

### Mechanisms of Se(IV) reduction in strain P10

**Fig 7:**
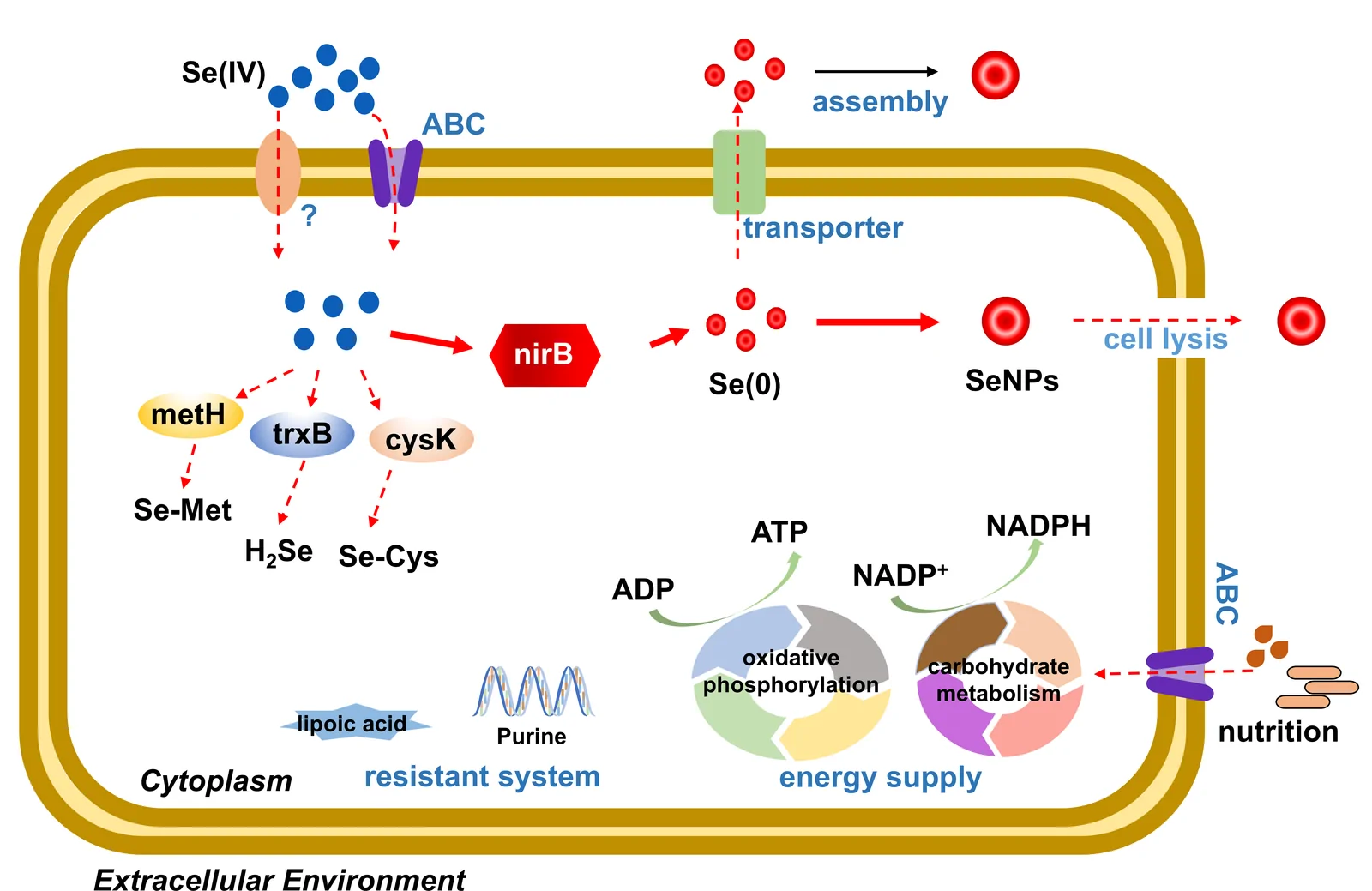
Proposed hypothetical model of Se(IV) reduction and SeNP formation in *P. polymyxa* P10: nitrite reductase (*nirB*), methionine synthase (*metH*), thioredoxin reductase (*trxB*), and cysteine synthase (*cysK*).

Based on transcriptomic and enzyme activity results, a hypothetical mechanism for Se(IV) reduction and SeNP formation in *P. polymyxa* P10 was proposed (Fig. 7). The process is suggested to involve three main stages: Se(IV) uptake, intracellular reduction, and nanoparticle formation/secretion. TEM images demonstrate that Se(IV) reduction primarily occurs intracellularly (Fig. 3), indicating that Se(IV) needs first to be transported into the cell. However, the specific Se(IV) uptake pathway was not directly identified in this study. Because 2,4-DNP showed only a partial and condition-dependent effect on NO_2_^–^ reduction by strain P10 (Table S2), the 2,4-DNP assay was not used to infer the role of nitrite transporters in Se(IV) uptake. Transcriptomic analysis showed that ABC transporter-related genes were enriched under Se(IV) exposure, suggesting that Se(IV) uptake may involve ABC transporters or other yet-unidentified transport systems. Therefore, ABC transporter-mediated Se(IV) uptake is proposed as a possible pathway rather than a confirmed mechanism.

Upon cytoplasmic entry, Se(IV) may trigger multiple metabolic responses. Together with the increased expression of *nirB*, the enzyme activity, and the inhibitory effect of nitrite on Se(IV) reduction, these data suggest that nitrite-reduction-related metabolism may be associated with Se(IV) transformation by *P. polymyxa* P10. Genes involved in selenocompound and sulfur metabolism, including *metH*, *trxB*, and *cysK*, may also participate in the cellular response to Se(IV) (30, 38). However, Raman and XPS analyses showed that Se(0) was the predominant reduction product, while no selenide-like products were detected (Fig. 4b and c). Therefore, the major functional contributors in *P. polymyxa* P10 are more likely associated with elemental Se formation rather than selenide production.

The Se(IV) reduction process may be energetically supported by enhanced carbohydrate metabolism and oxidative phosphorylation, which could generate essential reducing power (NADPH) and energy (ATP) for Se(IV) reduction and SeNP formation (29). The final stage may involve the release of SeNPs from the intracellular space to the extracellular environment. This can occur through multiple pathways: direct secretion, vesicular transport (facilitated by ABC transporters), or eventual cell lysis after prolonged cultivation (39).

### Repeated-batch Se(IV) reduction performance and stability

The repeated-batch Se(IV) reduction performance of strain P10 was evaluated over five consecutive 48 h cycles with an initial Se(IV) concentration of 1 mM (Fig. S4). In the first batch, Se(IV) was almost completely reduced within 36–48 h. Similar reduction profiles were observed in the subsequent four batches. Overall, strain P10 maintained near-complete Se(IV) reduction throughout all five cycles, indicating stable short-term performance under repeated-batch operation. This result provides preliminary evidence for the reusability of strain P10 under cyclic Se loading.

Under this solids-retaining repeated-batch mode, biomass and SeNP-containing solids were partially retained between cycles, which may have contributed to the stable reduction performance in later cycles. For residual Se(IV) analysis, samples were centrifuged before measurement, and the clarified supernatant was used to determine dissolved Se(IV), minimizing direct interference from retained solids. Nevertheless, this five-cycle experiment represents only short-term repeated-batch performance and should not be considered equivalent to long-term reactor validation. Longer-cycle operation, biomass/SeNP mass-balance analysis, and continuous-flow reactor tests are still needed to further evaluate the long-term engineering stability of strain P10.

### Conclusions

*P. polymyxa* P10 combines high Se(IV) tolerance, efficient Se(IV) reduction, and broad environmental adaptability, highlighting its promise for Se-containing wastewater treatment and SeNP recovery. Its stable performance in repeated-batch operation further indicates its feasibility for cyclic or semi-continuous treatment applications. Mechanistic analyses suggested that Se(IV) reduction in *P. polymyxa* P10 involves coordinated responses in nitrogen/sulfur-related metabolism, redox regulation, and energy supply. Nitrite reductase-associated or other molybdenum-dependent reductive pathways may participate in Se(IV) transformation, but their direct catalytic roles require further genetic and biochemical validation. These findings improve current understanding of microbial Se(IV) biotransformation and provide experimental support for the development of sustainable bioprocesses for Se-containing wastewater treatment and Se resource recovery.

## MATERIALS AND METHODS

### Isolation and characterization of Se(IV)-tolerant strain P10

Unless otherwise stated, all cultivation and Se(IV) reduction experiments were conducted under anaerobic conditions in an anaerobic chamber, with the media or sealed bottles deoxygenated by N_2_ flushing before incubation.

The Se(IV)-reducing microorganisms were isolated from sediment samples under anaerobic conditions in an anaerobic chamber. The sediment samples were collected at the Wuli Estuary, Huludao City, Liaoning Province, China (40°44′27″N, 120°55′55″E). The surface sediment contained an average total Se concentration of 11 mg/kg and had a pH of 7.35, indicating that this estuarine site represents a Se-enriched environment for isolating indigenous Se-transforming microorganisms. The modified LB medium used in this study contained yeast extract 5 g/L, tryptone 10 g/L, NaCl 10 g/L, and glucose 10 g/L. Fresh sediment was introduced into 50 mL of modified LB medium, supplemented with 5 mM Se(IV), and incubated for 7 days (40). Subsequently, the culture was diluted onto agar medium plates supplemented with 5 mM Se(IV). Se(IV)-reducing strain P10 was isolated based on its ability to produce red Se(0)-encrusted colonies on plates (12). The 16S rRNA gene of P10 was sequenced and compared to bacterial gene sequences previously published in the NCBI GenBank database.

The environmental adaptability of isolate P10 was evaluated by culturing it with various pH values (4–11), temperatures (15–45°C), and salt concentrations (1%–5% NaCl) in a modified LB medium. In different pH systems, 2-(N-morpholino) ethanesulfonic acid, 3-(N-morpholino) propanesulfonic acid, and N-Cyclohexyl-2-aminoethanesulfonic acid were used for pH 5 and 6 treatments, pH 7 and 8 treatments, and pH 9 and 10 treatments, respectively (1, 41). The initial bacterial density was 5.5 × 10^6^ cfu/mL. All experiments were performed in triplicate. Samples were taken periodically and diluted with 0.9% NaCl solution to determine the OD_600_ value.

To further evaluate Se(IV)-reducing activity under different environmental conditions, strain P10 was cultured in modified LB medium supplemented with 1 mM Se(IV) under the same pH, temperature, and salinity gradients described above. All experiments were performed in triplicate. Samples were collected at predetermined time points to determine residual dissolved Se(IV). Residual dissolved Se(IV) was quantified by hydride-generation atomic fluorescence spectrophotometry (AFS-8520). Liquid samples were centrifuged at 10,000 × *g* for 10 min to remove bacterial cells and particulate Se(0)/SeNPs. The clarified supernatants were diluted with 20% HCl and analyzed using KBH_4_ (20 g/L) and NaOH (3 g/L) as reducing agents. Se(IV) standards (0–50 μg/L) were used for calibration, and the instrument error was maintained within 3%. No digestion or oxidation step was applied; therefore, the measured values represent residual dissolved Se(IV), not total dissolved Se. Se(IV) reduction efficiency (%) = (initial Se(IV) concentration − residual dissolved Se(IV) concentration) / initial Se(IV) concentration × 100%.

### Evaluation of Se(IV) reduction capability

Isolate P10 was incubated under anaerobic conditions in media containing 1–50 mM Se(IV) at pH 7 and 37°C, with Se(IV) concentrations monitored throughout. High-dose reduction was tested in 100–1,000 mM Se(IV). The effects of anions (PO_4_^3–^, SO_4_^2–^, SO_3_^2–^, NO_3_^–^, and NO_2_^–^; 1 mM each) on 1 mM Se(IV) reduction were evaluated under the same anaerobic conditions. All experiments were performed in triplicate. At predetermined time points, liquid samples were collected, and Se(IV) reduction efficiency was determined as described above.

### Effect of inoculum size on bacterial growth and Se(IV) reduction

To identify an appropriate inoculation level for rapid Se(IV) reduction under engineering-relevant conditions, strain P10 was pre-cultured in modified LB medium to the exponential phase. Cultures were inoculated in modified LB medium supplemented with 1 mM Se(IV) using inoculation ratios of 0.5%, 1%, 2%, and 5% (vol/vol), respectively. All treatments were performed in triplicate biological replicates. At predetermined time points (0, 6, 12, 18, 24, 36, and 48 h), liquid samples were withdrawn aseptically for OD_600_ and residual Se(IV) concentration measurements.

### Morphological and chemical analysis of Se(IV) reduction products

Cultures of strain P10 were characterized after reducing Se(IV) to observe the location of the reduction products. Specifically, strain P10 was inoculated into modified LB medium and incubated for 24 h as a control. Parallel cultures were inoculated into the medium supplemented with 1 mM Se(IV) and incubated for 24 h and 48 h, respectively. After the designated incubation period, bacterial cultures were collected by centrifugation at 10,000 × *g* for 10 min. The pellets were then resuspended in a 2% glutaraldehyde solution and fixed overnight at 4°C. Following embedding in resin, sectioning, and staining, the samples were observed under an electron microscope (HT-7800, Hitachi High-Technologies, Tokyo, Japan).

After 48 h of Se(IV) reduction, the bacterial cultures were first centrifuged at 1,000 × *g* for 5 min to collect the extracellular SeNPs. The precipitates were washed three times with sterile deionized water and then freeze-dried. This fraction was designated as unpurified extracellular SeNPs and was used for FTIR analysis to evaluate the biomolecule-related functional groups associated with SeNPs before alkaline lysis and organic-solvent purification.

For total SeNP recovery, a parallel culture was centrifuged at 10,000 × *g* for 10 min to collect both bacterial cells and SeNP-containing precipitates. To release intracellular SeNPs, the precipitates were resuspended and boiled with 1 M NaOH at an elevated temperature for 20 min to facilitate cell lysis. The lysed material was then mixed with 1/2 volume of n-hexane to remove residual organic matter, and the resulting precipitate was washed and dried to obtain purified SeNPs. Subsequently, scanning electron microscopy (SEM-5000S, Guoyi Quantum Technology (Hefei) Co., Ltd., China) and energy dispersive spectroscopy (EDS) analyses were performed (42). The purified SeNPs were also characterized for composition by Raman spectroscopy (DXR microscope, Thermo Fisher, USA) and XPS (Axis Supra+, Shimadzu, Japan). FTIR spectra of both unpurified extracellular SeNPs and purified SeNPs were recorded using a Nicolet iS20 FTIR spectrometer (Thermo Fisher, USA) to compare changes in SeNP-associated organic functional groups before and after purification.

### Transcriptome analysis

Strain P10 was cultured in medium with or without 1 mM Se(IV). Samples for transcriptomic analysis were collected after 48 h of incubation, with three independent biological replicates for each treatment. The Se(IV)-treated cultures were first centrifuged at 500 × *g* to remove the precipitate, and all cultures were subsequently centrifuged at 10,000 × *g*. Total RNA was extracted from the cultures using the Trizol method (32). The quality of RNA was assessed with an Agilent 2100 Bioanalyzer (Agilent Technologies, USA), and the concentration was quantified using an ND-2000 spectrophotometer (NanoDrop Technologies, USA). Only RNA samples with RNA integrity number (RIN) values greater than 7.0 were used for library construction.

After rRNA removal, strand-specific RNA-seq libraries were constructed and sequenced on the Illumina platform using paired-end 150 bp sequencing. Raw reads were quality-filtered using fastp, with low-quality reads, adaptor sequences, and short reads removed. The clean reads were first aligned to the Rfam database to remove residual rRNA sequences and then mapped to the reference genome using Bowtie2. The reference genome used for read mapping was *Paenibacillus polymyxa* DSM 365 (NCBI RefSeq assembly accession GCF_000714835.1). The corresponding genome and annotation files were GCF_000714835.1_DSM365V1_genomic.fna.gz and GCF_000714835.1_DSM365V1_genomic.gff.gz, respectively.

Gene expression levels were quantified using RSEM. Transcript-level read counts were imported and summarized to gene-level read counts using the R package tximport, and normalized expression values were calculated as FPKM to account for gene length and sequencing depth. Differentially expressed genes (DEGs) analysis between the Se(IV)-treated and control groups was performed using edgeR based on gene-level read counts. The Benjamini–Hochberg method was used to control the false discovery rate (FDR). Genes with |log_2_ fold change| ≥ 1 and FDR ≤ 0.05 were considered significantly differentially expressed.

Gene Ontology (GO) and KEGG functional enrichment analyses of differentially expressed genes were performed using the R package clusterProfiler. Differentially expressed genes were used as the foreground gene set, and all expressed genes were used as the background gene set. Enrichment significance was assessed using a hypergeometric test, and *P*-values were adjusted for multiple testing using the Benjamini–Hochberg method. GO terms and KEGG pathways with adjusted *P*-values < 0.05 were considered significantly enriched.

### Enzyme inhibition experiments and enzyme activity assay

Three treatments were established to evaluate the effects of inhibitors on Se(IV) reduction and nitrite reductase activity: (i) a control without Se(IV) or inhibitor, (ii) a Se(IV)-treated group supplemented with 1 mM Se(IV), and (iii) a Se(IV) + sodium tungstate group supplemented with 1 mM Se(IV) and 10 mM sodium tungstate. Sodium tungstate is commonly used as an inhibitor of molybdenum cofactor-containing enzymes, including nitrate and nitrite reductases (34, 43). All treatments were inoculated with strain P10 and performed in triplicate. The concentration of Se(IV) was monitored throughout the cultivation process. The activity of nitrite reductase was measured using a Nitrite Reductase ELISA test kit (Solarbio, Beijing, China), and enzyme activity was quantified using an Infinite E Plex Microplate Reader (Tecan, Switzerland). The relative nitrite reductase activity in each treatment was calculated by normalizing the measured value to that of the control group. In addition, a separate positive-control assay was conducted to evaluate whether 2,4-DNP affected NO_2_^–^ reduction by strain P10. Strain P10 was incubated with 1 or 2 mM NO_2_^–^ in the presence or absence of 2 mM 2,4-DNP, and residual NO_2_^–^ concentrations were measured at predetermined time points.

### Repeated-batch Se(IV) reduction experiments

To evaluate the short-term operational stability and reusability of strain P10, repeated-batch tests were conducted under anaerobic conditions in N_2_-flushed sealed serum bottles containing modified LB medium supplemented with 1 mM Se(IV). Five consecutive 48 h cycles were performed (Cycle 1–Cycle 5). At the end of each cycle, the culture was allowed to settle briefly, and 90% (vol/vol) of the spent supernatant was replaced with fresh sterile medium containing 1 mM Se(IV), while the remaining slurry containing biomass and SeNP-containing solids was retained to initiate the next cycle. Samples were collected every 12 h, centrifuged at 10,000 × *g* for 10 min to remove cells and Se-containing solids, and the clarified supernatants were used to determine residual dissolved Se(IV) by AFS. All tests were performed in triplicate.

## Data Availability

The 16S rRNA gene sequence of *P. polymyxa* P10 is available in GenBank under accession number PZ418377. The RNA-seq raw reads generated in this study have been deposited in the NCBI Sequence Read Archive under BioProject accession number PRJNA1467252, with BioSample accession numbers SAMN60212871 and SAMN60212876.

## SUPPLEMENTAL MATERIAL

Supplemental material aem.00704-26-s0001.docx Fig. S1 to S4; Tables S1 and S2.

ASM does not own the copyrights to Supplemental Material that may be linked to, or accessed through, an article. The authors have granted ASM a non-exclusive, world-wide license to publish the Supplemental Material files. Please contact the corresponding author directly for reuse.
