# Supplementary material for: Bioconversion of selenite to selenium nanoparticles by Paenibacillus polymyxa P10: metabolic basis and potential for wastewater treatment
Source: Appl Environ Microbiol. 2026 Jul 17;92(8):e00704-26. doi: 10.1128/aem.00704-26 (PMC13488294; doi:10.1128/aem.00704-26)
Supplement: Supplementary file 1 [file aem.00704-26-s0001.docx]

**Supporting Materials**

**Bioconversion of selenite to selenium nanoparticles by *Paenibacillus polymyxa* P10: metabolic basis and potential for wastewater treatment**

Jiaxing Zhou ^a,b^, Changpeng Sang ^a^, Zhunan Liu ^c^, Weiming Li ^b,^*, Yanchen Sun ^d^, Jiaxi Zhang ^b^, Shaofeng Wang ^a^, Xiangfeng Zeng ^b,^*

^a^ Key Laboratory of Industrial Ecology and Environmental Engineering (Ministry of Education), School of Environmental Science and Technology, Dalian University of Technology, Dalian 116024, China

^b^ Institute of Applied Ecology, Chinese Academy of Sciences, Shenyang, Liaoning 110016, China

^c^ Liaoning Geology and Mineral Resources Institute Co., Ltd., Shenyang 110032, China

^d^ Department of Marine Chemistry and Geochemistry, Woods Hole Oceanographic Institution, Woods Hole, Massachusetts 02543, United States

*Corresponding author.

E-mail address: [liwm@iae.ac.cn(W.Li)](mailto:liwm@iae.ac.cn(W.Li))

# Figure supplement

**Fig. S1.** Morphological and phylogenetic characterization of *P. polymyxa* strain P10: (a) Gram-stained microscopy (×1000); (b) Visual appearance of bacterial culture after 24 h incubation in modified LB medium; (c) Neighbor-joining phylogenetic tree based on 16S rRNA gene sequences.

**Fig. S2.** Growth of *P. polymyxa* P10 in modified LB medium under varying conditions: (a) Different pH levels at 37 °C; (b) Different temperatures at pH 7; (c) Different salinities at pH 7 and 37 °C. Data are shown as mean ± SD, n = 3.

**Fig. S3.** Effect of inoculum size on bacterial growth and Se(IV) reduction by strain P10: (a) Time-dependent changes in biomass concentration (OD_600_) at different inoculum sizes (0.5%–5%, v/v); (b) Corresponding Se(IV) reduction efficiency during incubation. Data are shown as mean ± SD, n = 3.

**Fig. S4.** Repeated batch Se(IV) reduction performance of strain P10. Time-dependent Se(IV) reduction efficiency during five consecutive batch cycles, each operated for 48 h with an initial Se(IV) concentration of 1 mM. Data are shown as mean ± SD, n = 3.

# Table supplement

**Table S1.** Representative differentially expressed genes involved in major Se(IV)-responsive pathways in *P. polymyxa* P10.

| **Pathway** | **Protein** | **Gene name** | **Description** | **Regulation** | **log_2_FC** | **FDR** |
| --- | --- | --- | --- | --- | --- | --- |
| Nitrogen metabolism | WP_040103386.1 | nirB | nitrite reductase | Up | 1.28 | 1.31E-05 |
|  | WP_040103387.1 |  | assimilatory nitrate reductase catalytic subunit NasC | Down | -1.20 | 1.48E-11 |
|  | WP_016324613.1 | narH | nitrate reductase subunit beta | Down | -1.31 | 1.23E-03 |
| Sulfur metabolism | WP_025678913.1 | cysC | adenylyl-sulfate kinase | Up | 1.66 | 1.50E-23 |
|  | WP_013368700.1 | cysK | cysteine synthase A | Up | 1.18 | 2.47E-31 |
|  | WP_040101786.1 | metA | homoserine O-succinyltransferase | Down | -1.13 | 5.13E-06 |
| Selenocompound metabolism | WP_040102863.1 | trxB | thioredoxin-disulfide reductase | Up | 2.73 | 1.04E-212 |
|  | WP_040103577.1 | metH | methionine synthase | Down | -1.11 | 1.34E-24 |
|  | WP_017428578.1 | metE | 5-methyltetrahydropteroyltriglutamate--homocysteine S-methyltransferase | Down | -1.61 | 3.10E-30 |
| ABC transporters | WP_040101757.1 |  | iron-siderophore ABC transporter substrate-binding protein | Up | 1.94 | 6.18E-08 |
|  | WP_013309592.1 | pstA | phosphate ABC transporter permease PstA | Up | 1.64 | 1.21E-23 |
|  | WP_013370343.1 | pstC | phosphate ABC transporter permease subunit PstC | Up | 1.64 | 2.77E-33 |
|  | WP_016819624.1 | pstB | phosphate ABC transporter ATP-binding protein PstB | Up | 1.49 | 8.35E-15 |
|  | WP_040102551.1 | nikA | nickel ABC transporter substrate-binding protein | Up | 1.49 | 4.39E-02 |
|  | WP_013369092.1 |  | methionine ABC transporter permease | Up | 1.42 | 8.64E-03 |
| Oxidative phosphorylation | WP_013372928.1 | cyoE | heme o synthase | Up | 1.79 | 1.00E-48 |
|  | WP_013373647.1 |  | NAD(P)/FAD-dependent oxidoreductase | Up | 1.07 | 1.33E-41 |
|  | WP_007432750.1 | atpE | F0F1 ATP synthase subunit C | Down | -1.12 | 3.05E-04 |
|  | WP_040102703.1 | atpF | F0F1 ATP synthase subunit B | Down | -1.27 | 6.22E-19 |

**Table S2.** Effect of 2 mM 2,4-DNP on NO_2_^–^ reduction by *P. polymyxa* P10 under different initial NO_2_^–^ concentrations.

| Treatments | NO_2_^–^ concentration (mM) | | |
| --- | --- | --- | --- |
| NO_2_^–^ + 2,4-DNP (mM) | 0 h | 24 h | 48 h |
| 1+0 | 1.03±0.07 | 0 | 0.02±0.03 |
| 1+2 | 1.01±0.05 | 0 | 0 |
| 2+0 | 2.05±0.07 | 0.26±0.01 | 0 |
| 2+2 | 2.01±0.09 | 0.73±0.04 | 0.53±0.03 |
